# Multi-band high-frequency antenna for satellite, automotive radar, and 6G communication

**DOI:** 10.1038/s41598-025-16217-9

**Published:** 2025-08-19

**Authors:** Swati Varun Yadav, Manish Varun Yadav, Dinesh Yadav, Gaurav Kumar Soni

**Affiliations:** 1 Department of Instrumentation and Control Engineering, Manipal Institute of Technology, Manipal Academy of Higher Education, Manipal, Karnataka 576104 India; 2 Department of Aeronautical and Automobile Engineering, Manipal Institute of Technology, Manipal Academy of Higher Education, Manipal, Karnataka, 576104 India; 3 Department of Electronics and Communication Engineering, Manipal University Jaipur, Jaipur, Rajasthan India

**Keywords:** Satellite, Automotive radar, 6G communication, Impedance bandwidth, High efficiency, Engineering, Physics

## Abstract

This paper presents the design and development of a compact multi-band high-frequency antenna tailored for millimeter-wave applications, particularly within the 6G frequency range. The antenna features a small footprint of 10 × 12 × 1.5 mm^2^ and is designed using advanced electromagnetic simulations in CST Microwave Studio. Fabrication was carried out on an FR4 substrate, selected for its favorable properties at high frequencies. The antenna demonstrates an exceptionally wide impedance bandwidth of approximately 166%, covering a broad frequency range from 9.1 GHz to 100 GHz with a central frequency near 45.45 GHz. It exhibits stable radiation characteristics across the operating band, achieving a peak gain of 7.95dBi and an overall efficiency of 85%. Its miniaturized form factor and broad operational range make it a strong candidate for a wide spectrum of applications, including X-band radar, Ku-band satellite communications, K-band sensing, Ka-band 5G systems, V-band short-range wireless, W-band automotive radar, and future technologies such as 6G and security imaging systems.

Figure 1 presents a comprehensive overview of the proposed methodology. It starts with the simulation of the antenna design using CST software to evaluate its theoretical performance. The next segment highlights the antenna fabrication process conducted in the Antenna Laboratory, where a chemical etching technique is employed to realize the physical structure of the antenna. This is followed by testing and measurement of the fabricated antenna to assess its real-world performance. The final part of the figure compares the simulation results with the measured data to verify the accuracy and reliability of the proposed design.

**Fig. 1:**
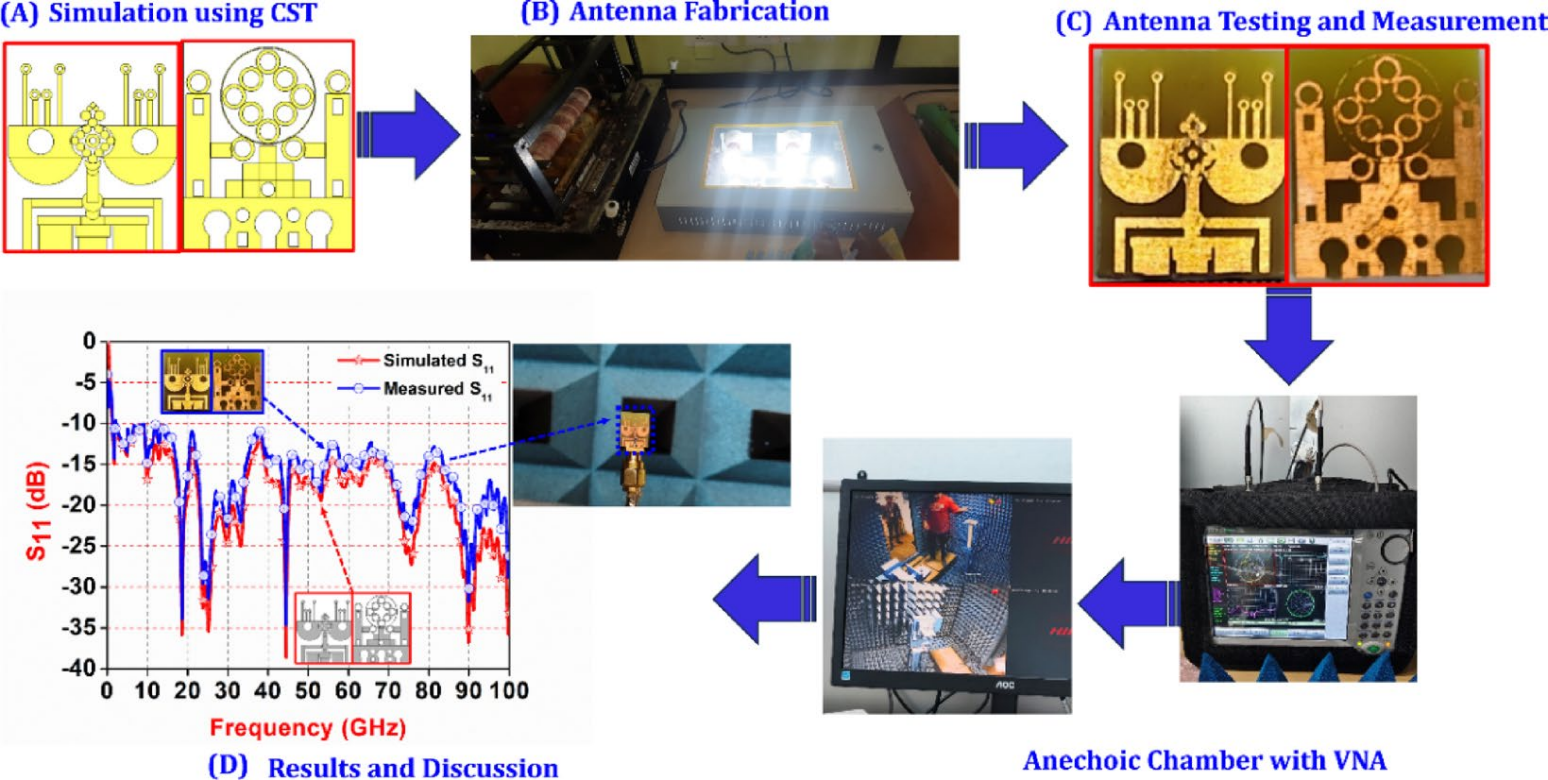
A graphical representation of the proposed methodology: (**A**) Simulation of the antenna using CST software; (**B**) Illustration of the Antenna Laboratory fabrication setup; (**C**) Depiction of the fabricated antenna design along with its testing and measurement process; (**D**) Comparison of simulation and experimental results.

## Introduction

In the current era of rapid technological advancements, the demand for fast and dependable wireless communication systems has become increasingly critical. To address these needs, antenna designs must be carefully optimized for specific frequency bands. This work presents a compact antenna specifically engineered for the millimeter-wave 6G frequency range, with its performance rigorously analyzed through simulations conducted using CST Microwave Studio.

Recent developments in microstrip patch antenna technology have led to a variety of novel and high-performance designs. For instance, Mishra et al. (2021) examined key performance parameters of microstrip patch antennas, focusing on different structural configurations and techniques aimed at improving bandwidth and overall efficiency^1^. Similarly, to support the growing requirements of high-frequency applications, Przesmycki et al. (2020) introduced a wideband microstrip antenna intended for 5G communications at the 28 GHz band^2^. Similarly, Imran et al. (2018) focused on millimeter-wave microstrip patch antennas tailored for 5G mobile communication applications, emphasizing the importance of customized design considerations for optimal deployment^3^. Additionally, Khidre et al. (2013) introduced a wideband dual-beam U-slot microstrip antenna, showcasing its potential for dual-beam operation and broad frequency coverage^4^.

Expanding on this foundation, our research undertakes an in-depth analysis of symmetrically shaped antennas, examining factors such as radiation pattern, resonating band, antenna size, and dielectric material composition. Moreover, we investigate methods to enhance the bandwidth of microstrip patch antennas, drawing upon concepts from physics and circuit theory to maximize performance across various operational contexts^5–8^. Transitioning to the literature on millimeter-wave and terahertz frequency bands, Akyildiz, Han, and Nie (2018) offer compelling insights into addressing distance challenges inherent in these high-frequency ranges, providing valuable guidance for optimizing millimeter-wave and terahertz communications^9^. Additionally, Akyildiz, Jornet, and Han (2014) explore the promise of the terahertz band as the next frontier in wireless communications, outlining key prospects and challenges in utilizing this spectrum for cutting-edge applications^10^.

Furthermore, Han and Chen (2018) contribute insightful information about the unique qualities and challenges associated with terahertz propagation, laying the groundwork for the development of more robust and efficient wireless networks in the terahertz range^11^. In conclusion, this study provides a comprehensive review of the evolution of microstrip patch antennas and their role in contemporary wireless communication systems. By synthesizing recent developments and innovative designs in antenna technology, our objective is to support ongoing efforts to enhance wireless connectivity and facilitate revolutionary advancements in telecommunications.

## Geometry and design structure

**Fig. 2:**
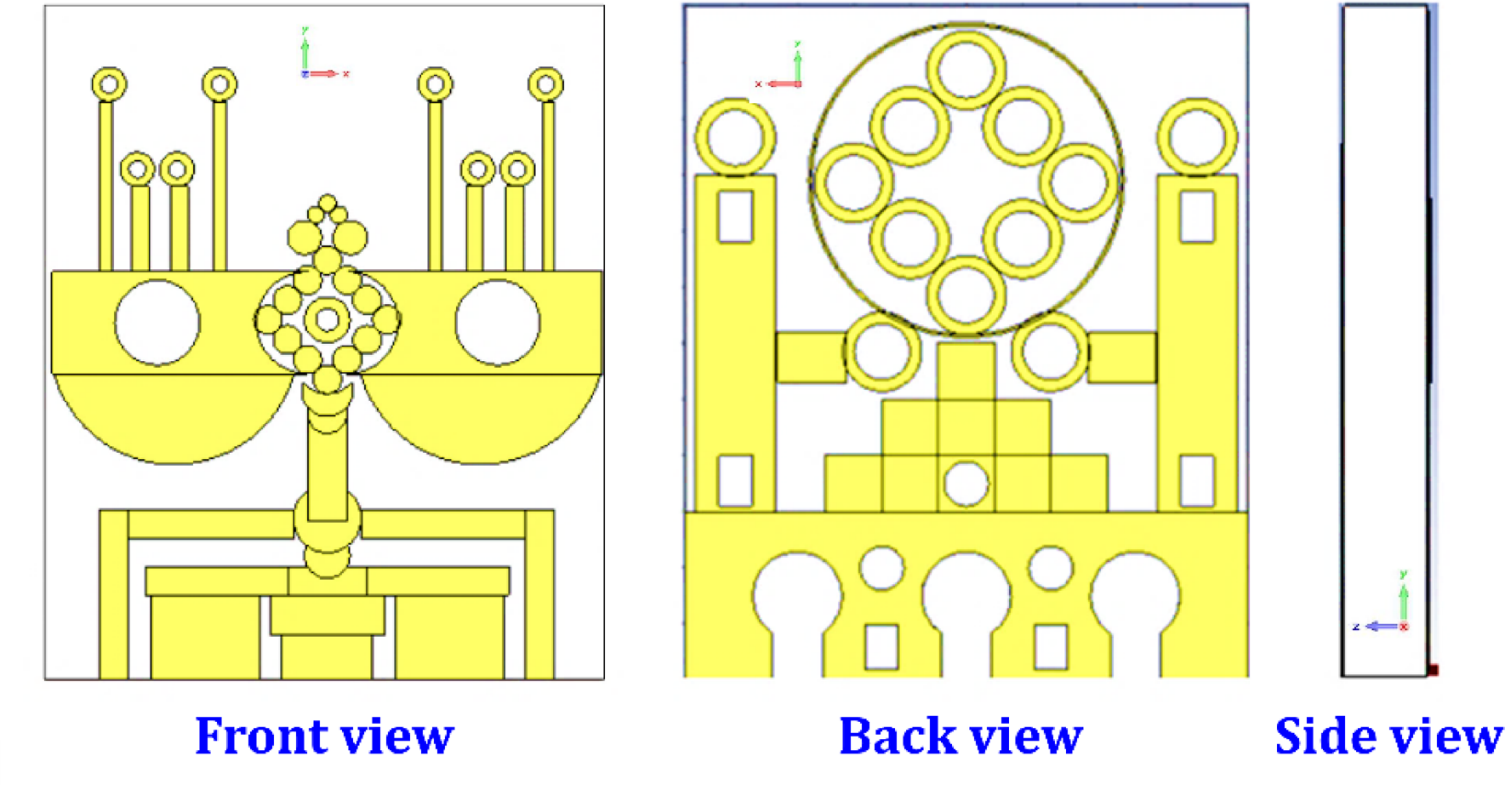
Visual representation of the proposed.

The Fig. 2 illustrates the final design of the proposed antenna, showcasing its front, back, and side views with detailed structural features that embody a highly optimized layout aimed at achieving advanced performance characteristics such as wideband operation, multiband resonance, and enhanced radiation properties. The front view reveals the primary radiating elements, including a central circular array of small ring-shaped resonators arranged in a symmetrical floral or diamond pattern around the feed point, which supports multiple resonances. Flanking this are two large semi-circular patches with circular slots acting as parasitic elements to boost bandwidth and gain. Vertical stubs with small ring resonators further aid tuning and impedance matching, while the feedline and matching network beneath highlight a complex design for optimal energy transfer. The back view highlights the defected ground structure (DGS), featuring a large circular ring array aligned with the front’s symmetry and a pyramid-like pattern of square and circular slots to control surface currents, suppress unwanted modes, and improve radiation efficiency. Additional vertical stubs and rectangular slots contribute to current flow management and may support polarization diversity. The side view depicts the layered structure of the antenna, illustrating the substrate thickness and spatial arrangement between the front radiating elements and the back ground plane, confirming its planar form factor. Together, the front and back views present a sophisticated antenna design tailored for efficient signal transmission and fine-tuned electromagnetic performance, making it well suited for modern high-frequency applications such as 5G, IoT, and satellite communication systems.

**Fig. 3:**
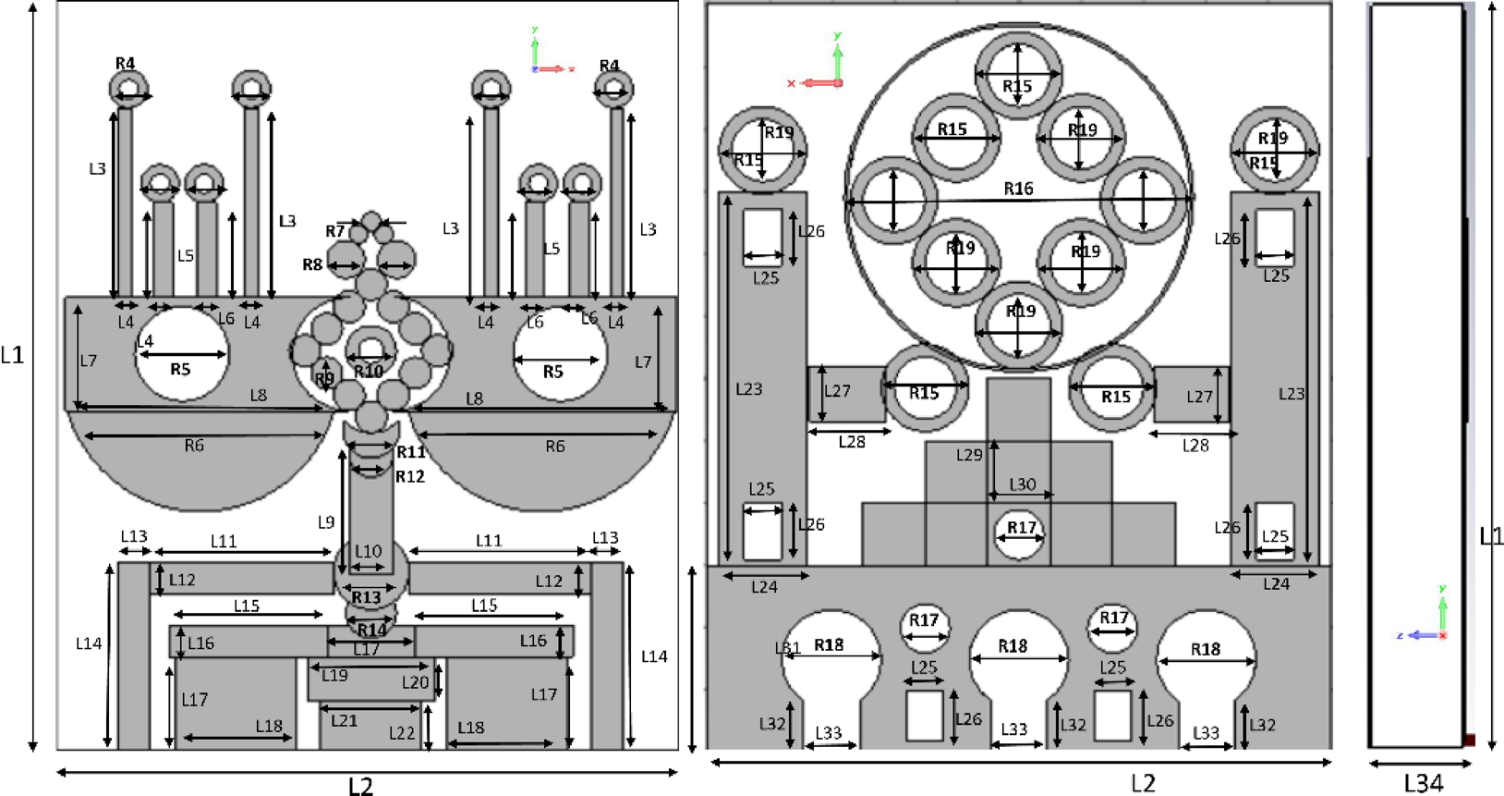
Conceptual design of an antenna.

Figure 3 illustrates the antenna’s structural dimensions, including its length (‘L2’), width (‘L34’), and height (‘L1’). This section thoroughly explores the design parameters that define the radiating structure. At the heart of the configuration is a diamond-shaped arrangement of twelve circular elements, labeled ‘R9’. On both sides of this central layout are two rectangular strips with dimensions ‘L7’ and ‘L8’. A circular cut-out, marked ‘R5’, is integrated into the primary rectangular patch, and a semicircular component, denoted as ‘R6’, is positioned at the bottom. A microstrip connection, measured by ‘L9’ and ‘L10’, links the diamond-shaped array to another circular section labeled ‘R13’. This circular patch is complemented by two bench-like rectangular shapes with sizes ‘L11’ × ‘L12’ and ‘L14’ × ‘L13’. In Table 1 represent the parameters and values of the proposed antenna.

**Table 1:** Parameters and values of the proposed antenna.

Parameters	L1	L2	L3	L4	L5	L6	R1	R2	R3
Values	12	10	3	0.2	1.5	0.3	1.4	0.9	0.4
Parameters	R8	R9	R10	R11	R12	L9	L10	R13	L11
Values	0.8	0.25	0.4	0.45	0.35	1.9	0.7	0.6	2.9
Parameters	R14	L17	L18	L19	L20	L21	L22	R15	R16
Values	0.4	1.5	1.9	2	0.7	1.6	0.8	0.7	2.8
Parameters	L29	L30	R17	L31	R18	L32	L33	R19	L34
Values	1	1	0.4	3	0.8	0.9	0.9	0.5	1.5
Parameters	R4	R5	L7	L8	R6	R7	L23	L24	L27
Values	0.3	0.75	1.8	4.5	2.2	0.15	6	1.4	0.9
Parameters	L12	L13	L14	L15	L16	L17	L25	L26	L28
Values	0.5	0.5	3	2.5	0.5	1.4	0.6	0.9	1.2

The backside of the antenna features a distinctive hollow circular design marked ‘R16’, which houses eight cylindrical elements with inner and outer radii labeled ‘R15’ and ‘R19’. Beneath this structure is a formation of nine rectangular elements arranged in a pyramid-like fashion, each with dimensions ‘L29’ × ‘L30’. On both sides are supporting rectangular pillars (‘L23’ × ‘L24’), each featuring two rectangular slots of dimensions ‘L26’ × ‘L25’. The antenna was manufactured using a highly accurate chemical etching technique, chosen for its precision and consistency. Careful calibration of fabrication parameters ensured uniformity and quality throughout production. A thorough post-fabrication inspection process was implemented to identify and correct any imperfections.

## Evolution of the antenna

**Fig. 4:**
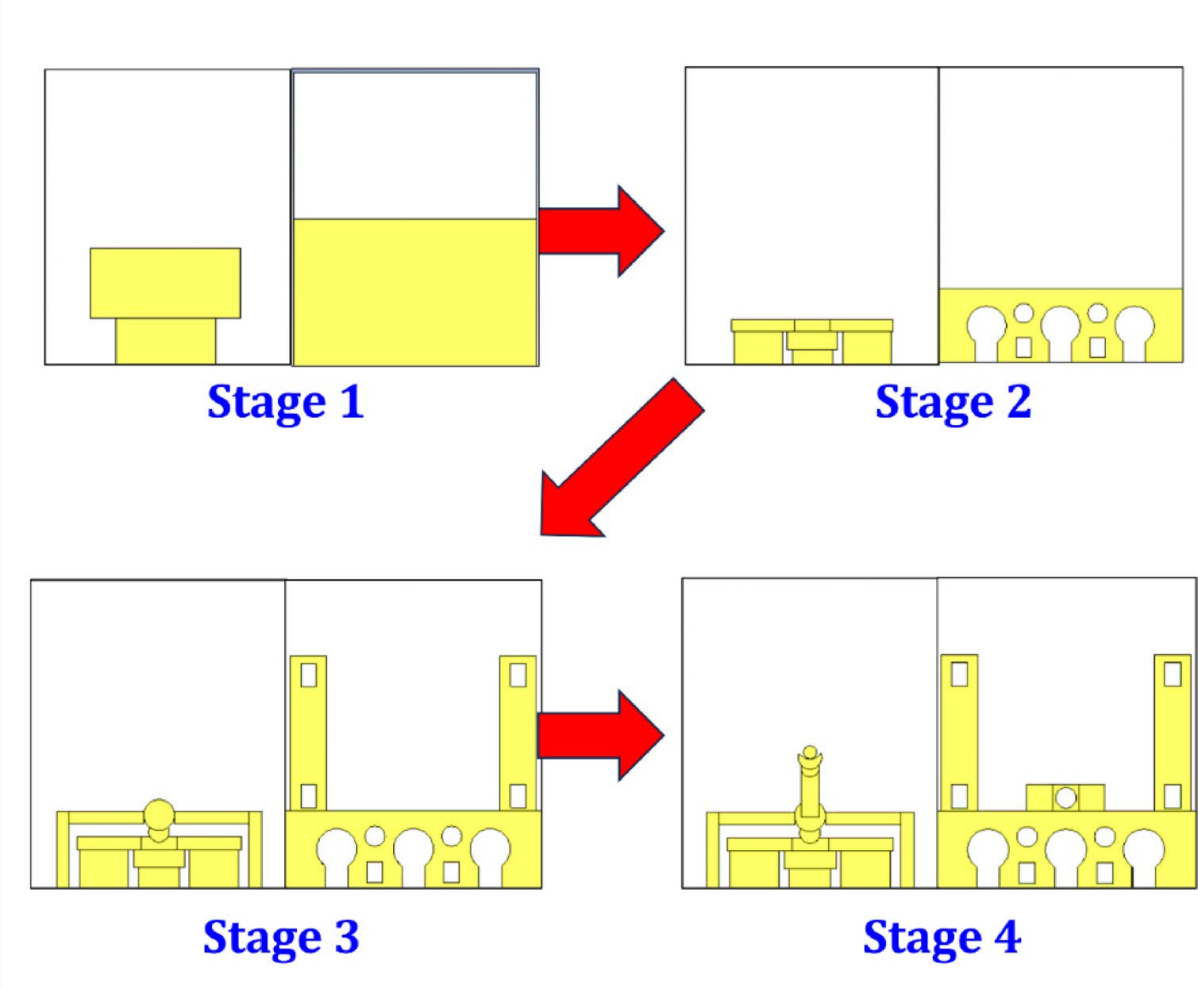
Antenna evolution from Stage 01–04.

The antenna design progresses through four distinct developmental phases, as illustrated in Fig. 4, with each stage incorporating specific structural enhancements to boost overall performance. In the initial phase (Stage 1), the design consists of a basic rectangular patch on the top surface paired with a partial ground plane beneath. This configuration serves as the starting point, generally supporting single-frequency operation with limited bandwidth and moderate gain. In Stage 2, the structure becomes more intricate with modifications to both the radiating patch and the ground plane. The feed line is extended and reconfigured, while a combination of circular and rectangular slots is introduced into the ground layer. These changes are implemented to improve impedance matching, broaden bandwidth, and enable multiband performance by utilizing a defected ground structure (DGS).

In Stage 3, the radiating element is further refined with additional slots, stepped sections, and a circular stub, enhancing the control over surface current distribution. Vertical arms are also added to the structure, providing greater tuning flexibility and helping to support dual- or triple-band resonance. The ground plane retains the previously introduced slots, maintaining its role in influencing the antenna’s radiation characteristics. Finally, Stage 4 presents the fully optimized antenna configuration. The central feed is further refined, possibly using a coaxial or probe-fed mechanism, and the radiating patch includes both circular and square slots arranged symmetrically. This final design stage is aimed at maximizing overall antenna performance, including wideband or multiband operation, improved return loss, enhanced radiation efficiency, and the potential for polarization diversity. The design evolution clearly demonstrates a methodical approach to achieving a compact, high-performance antenna suitable for modern wireless applications.

**Fig. 5:**
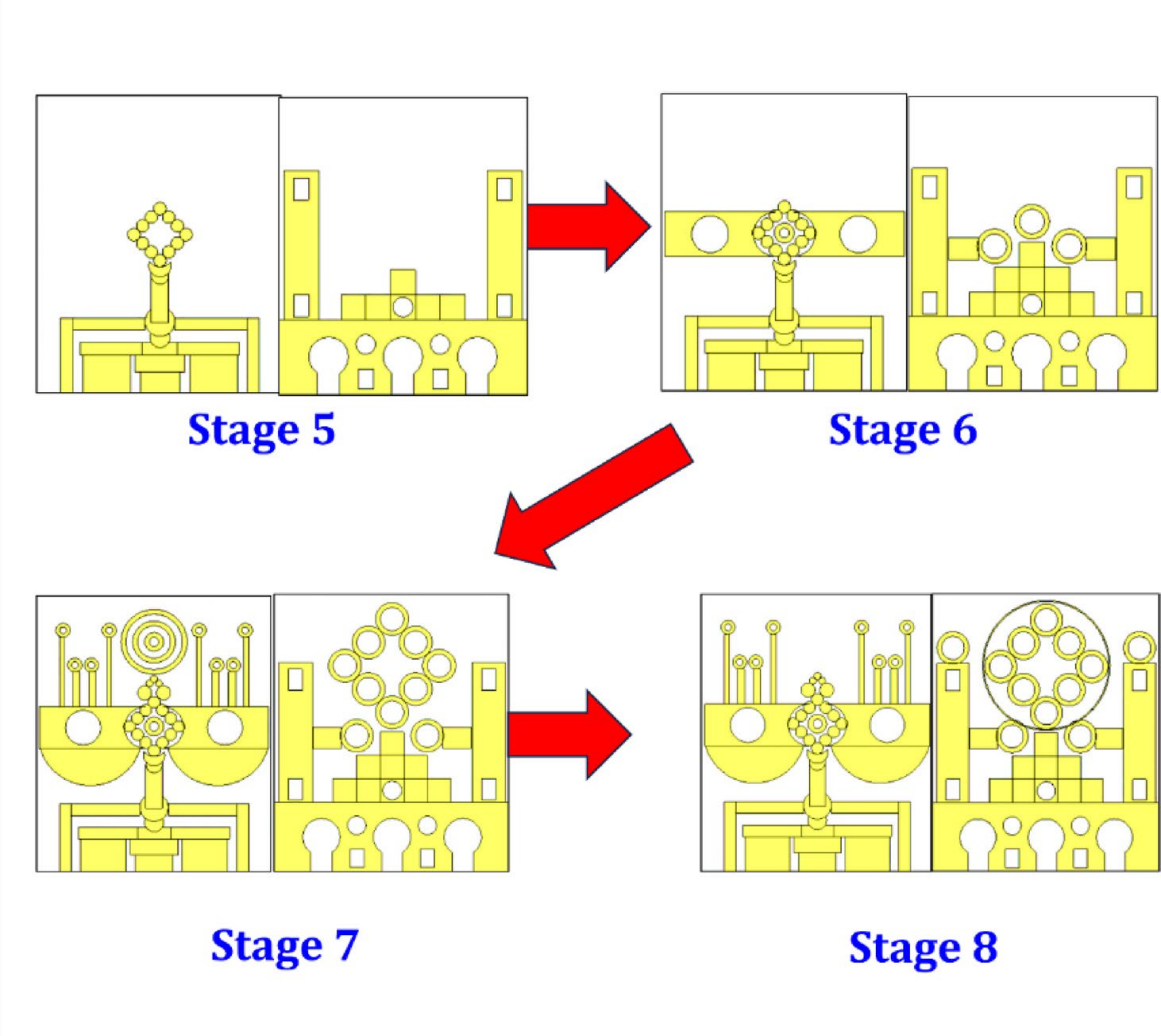
Antenna evolution from Stage 05–08.

The progression from Stage 5 to Stage 8, as shown in Fig. 5, in the proposed antenna design illustrates a clear move toward increasing structural complexity and performance refinement, especially in terms of bandwidth enhancement, multiband functionality, and radiation characteristics. In Stage 5, the antenna introduces a diamond-shaped radiating structure composed of circular stubs arranged symmetrically around the central feed. This intricate design significantly alters the current distribution, likely introducing additional resonant frequencies. The ground plane continues to feature circular and rectangular slots, aiding in bandwidth tuning and improving impedance matching.

Advancing to Stage 6, the radiating element is further evolved into a floral or petal-like configuration, integrating more circular features and branching structures. On the ground plane side, a symmetrical pyramid-like stacking of square and circular elements is introduced, enriching the defected ground structure. These modifications are aimed at expanding the effective surface area for current flow and introducing additional resonant paths, contributing to wider bandwidth and improved multiband operation.

In Stage 7, the antenna becomes considerably more elaborate. The top layer now includes concentric ring structures and extended vertical stubs, contributing to improved gain and potentially circular polarization. The ground plane incorporates a large cluster of circular resonators in a hexagonal arrangement, enhancing coupling and further enabling multiband characteristics. This stage marks a significant shift toward a highly engineered metamaterial-inspired design.

Finally, in Stage 8, the proposed antenna reaches its most optimized form. The radiating structure features a highly symmetrical, circular array of ring resonators around a central feed, which improves radiation uniformity and bandwidth even further. The ground plane mirrors this refinement, incorporating circular and square elements that support both impedance control and miniaturization. This final configuration is likely designed for high-performance applications such as 5G, IoT, or satellite communication systems, offering wide bandwidth, multiple resonant bands, and efficient radiation properties. Overall, the evolution from Stage 5 to Stage 8 highlights a meticulous and effective approach to modern antenna design, balancing complexity with performance gains.

**Fig. 6:**
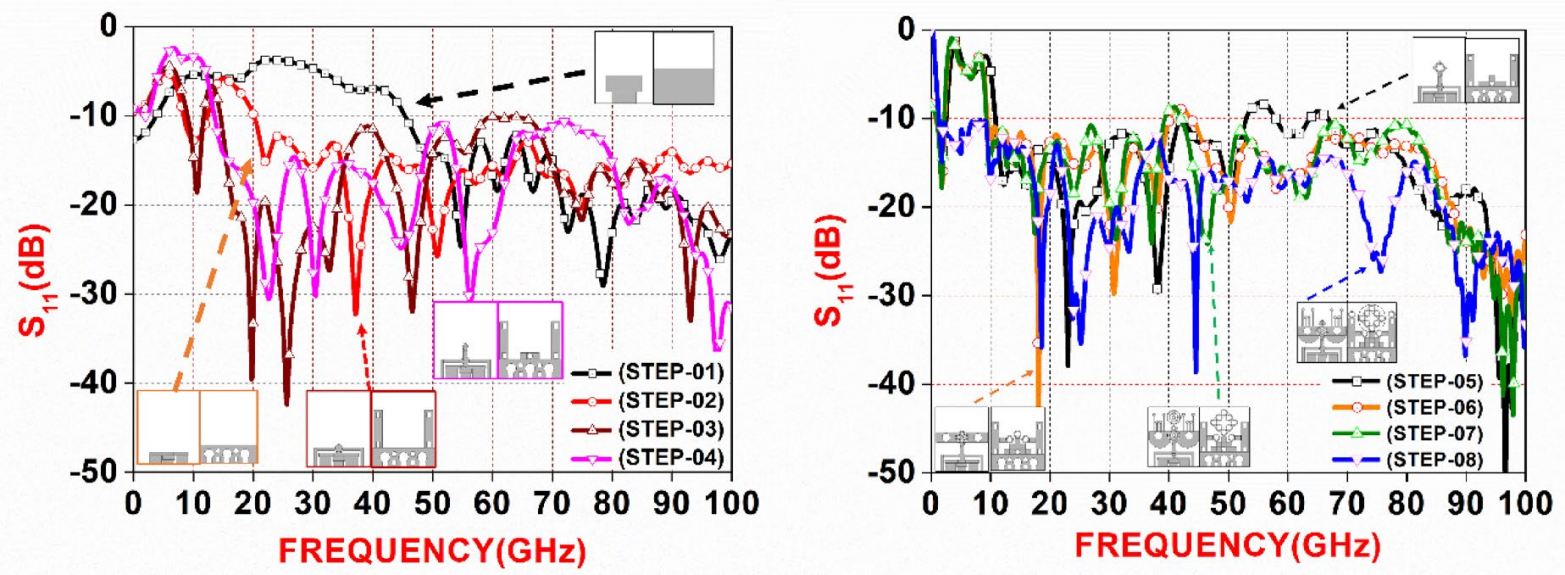
All stages S_11_ parameter, (**a**) stage 01–04, (**b**) stage 05–08.

Figure 6 shows all stages of antenna evolution. The antenna design evolves through four progressive stages to enhance its performance and impedance bandwidth. Initially, a simple rectangular patch (6 × 2.5 mm^2^) with a microstrip feedline (4 × 2 mm^2^) demonstrates limited resonance and poor impedance matching beyond 30 GHz, as reflected by a high reflection coefficient (S_11_ > − 10 dB). In the second phase, two rectangular patches are added along the ground plane, improving surface current distribution and supporting a broader single-band response from around 32 GHz to 100 GHz, though lower frequency matching remains moderate. The third stage introduces three bulb-shaped slots on the rear rectangular strip, significantly boosting resonance in the 46–100 GHz range with deep nulls (S_11_ < − 20 dB), indicating strong multi-band operation. In the final configuration, a circular patch is added at the front, accompanied by bench-like rectangular features and pillar-shaped slots on the backside, resulting in substantial impedance improvement and ultra-wideband coverage from 20 GHz to 100 GHz with consistent S_11_ below − 10 dB.

The antenna design progresses through several enhancement stages, each aimed at improving resonance and bandwidth. In STEP-05, a base structure comprising multiple rectangular and circular patches is established, achieving moderate resonance between 18 GHz and 95 GHz. STEP-06 introduces eleven circular patches arranged in a diamond pattern on the front and adds three small rectangular patches atop the central element, extending performance from 15 GHz to 100 GHz. In STEP-07, additional rectangular shapes with circular slots are positioned near the diamond structure, while three more rectangular patches form a pyramid-like arrangement at the rear, lowering the resonance threshold to 12 GHz. STEP-08 finalizes the design by incorporating two semicircular patches at the base, four vertical pole-like structures, and three cylindrical elements near the pyramid structure. A carefully optimized ground plane and an enclosing circular patch around the rhombus-like formation further enhance resonance, enabling ultra-wideband performance from 9.1 GHz to 100 GHz.

## Parameter study

**Fig. 7:**
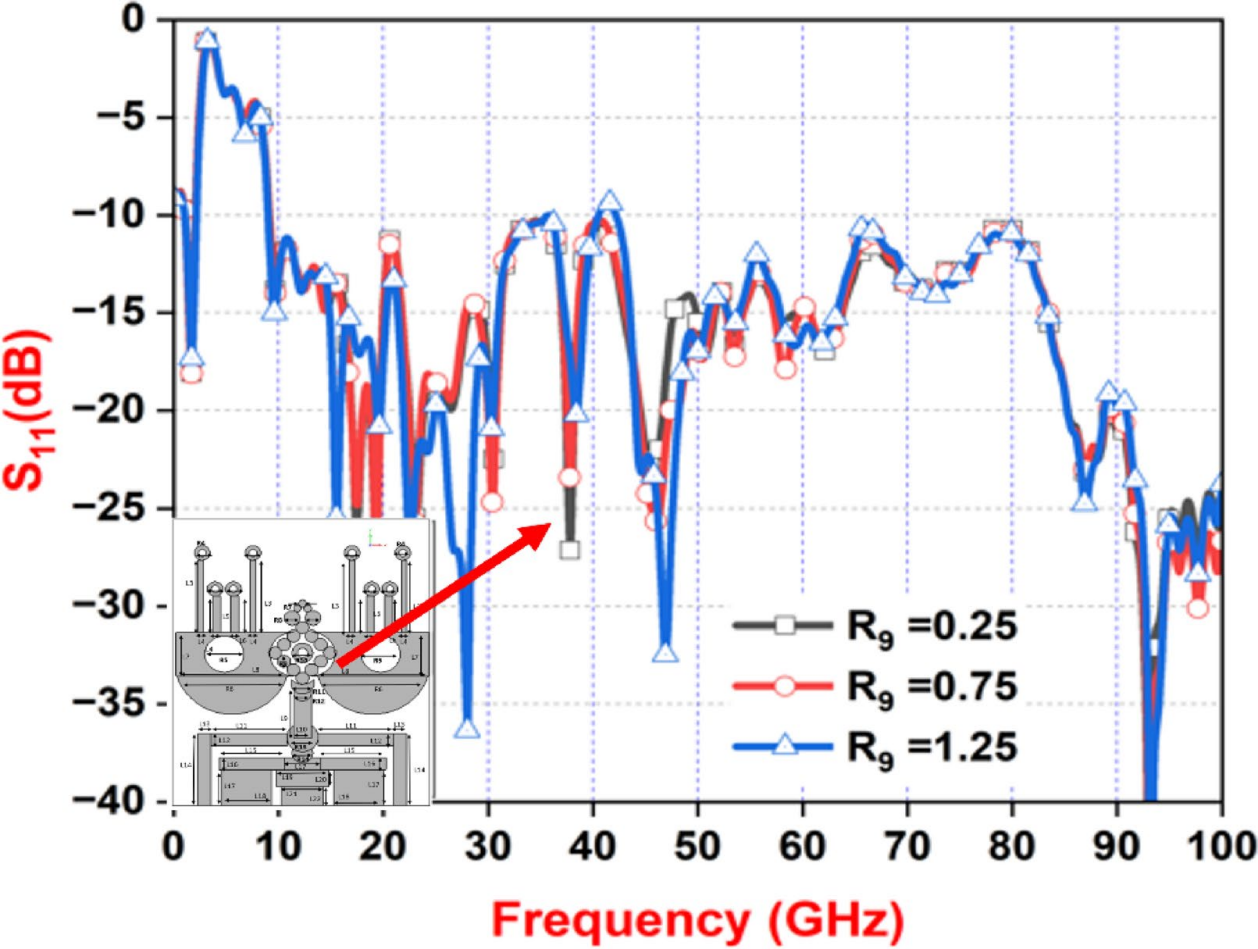
Simulated parameter Sweep ‘R9’.

As part of our parameter study, we next examined R9 in Fig. 7, which defines the diameter of the parasitic circular element positioned at the front of the antenna. This dimension was varied between 0.25 mm and 1.25 mm to assess its influence on antenna behavior. The results indicated that a diameter of 0.25 mm delivered the most favorable performance, successfully exciting both lower- and higher-order resonance modes across a broad frequency range of 9.1 GHz to 100 GHz. Moreover, this configuration maintained the S11 value a key indicator of antenna return loss below − 10 dB throughout the spectrum. Figure 7 clearly illustrates the performance improvement achieved through this optimization.

**Fig. 8:**
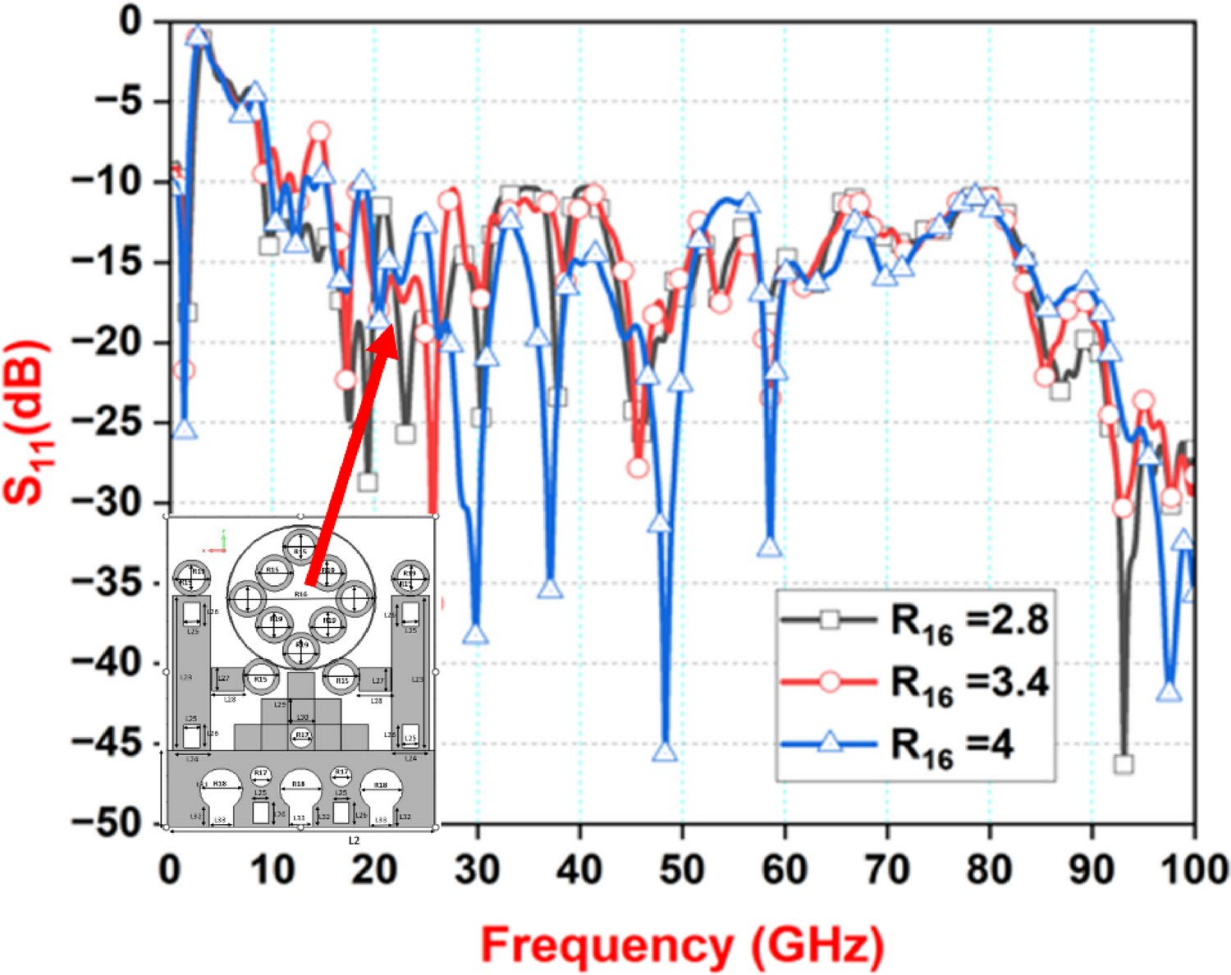
Simulated parameter Sweep ‘R16’.

We also analyzed the diameter of the R16 circular structure located on the antenna’s backplane. To determine the most effective configuration, we tested various heights within the range of 2.8–4 mm. Through detailed evaluation, we found that a height of 2.8 mm provided the most favorable results, particularly by enhancing impedance matching at lower frequencies, around 48 GHz. The outcomes of this analysis are presented in Fig. 8. These results will be elaborated upon in the subsequent section. Adjusting this parameter played a critical role in optimizing the antenna’s performance and was a key step in refining the overall design.

## Measurements and simulated results

**Fig. 9:**
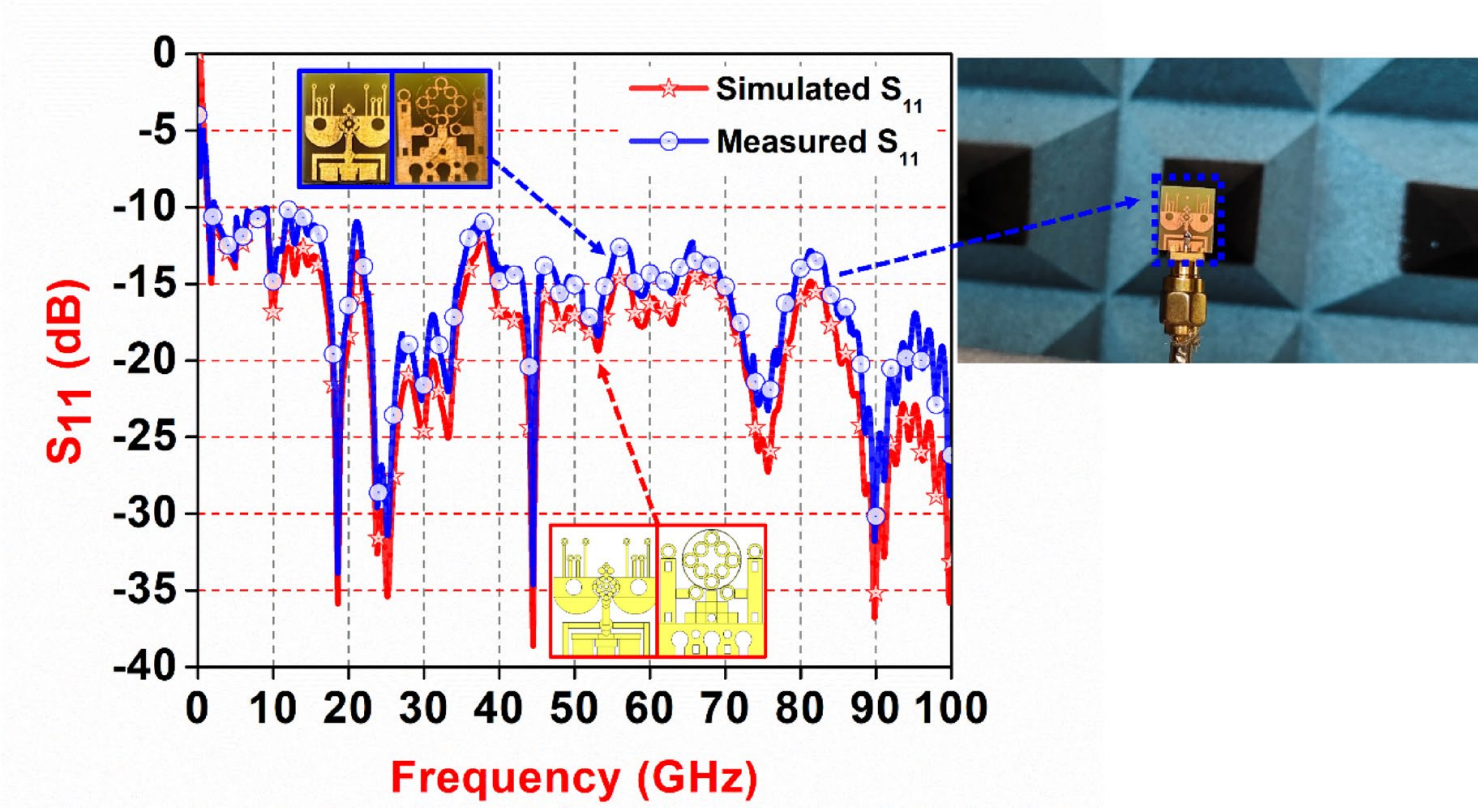
Comparison between simulated and measured S_11_ (reflection coefficient).

To validate the real-world performance of the proposed antenna, measurements were carried out on the fabricated prototype using a Vector Network Analyzer (VNA) inside an anechoic chamber. The Fig. 9 compares the simulated and measured S_11_ results, where the red curve represents the simulation and the blue curve shows the measured data. Both results demonstrate a wide impedance bandwidth of 166%, effectively covering the 9.1 GHz to 100 GHz frequency range with a center frequency at 45.45 GHz. The return loss remains below − 10 dB across the entire band, indicating good impedance matching. The strong agreement between simulation and measurement confirms the accuracy and reliability of the antenna design, with only minor deviations attributed to manufacturing tolerances and measurement setup.

**Fig. 10:**
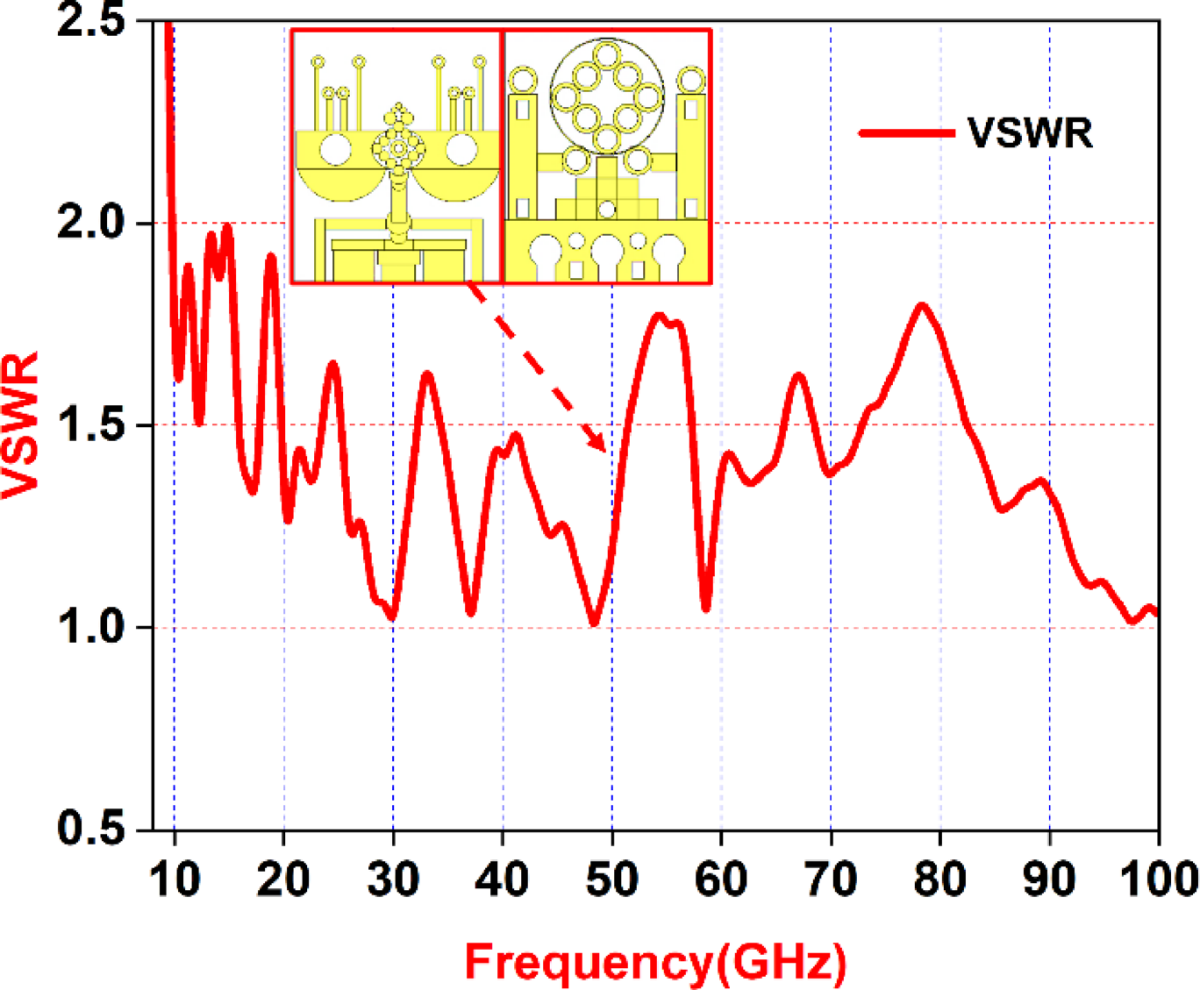
The VSWR curve of the proposed antenna.

Figure 10 illustrates the Voltage Standing Wave Ratio (VSWR), a key indicator used to assess antenna performance. VSWR represents the ratio between the maximum and minimum voltage along a transmission line and is essential in determining how effectively power is transferred from the antenna to the system. A decreasing VSWR value across the frequency range of 9.1 GHz to 100 GHz indicates better impedance matching and improved efficiency.

The graph in Fig. 10 highlights the VSWR behavior of the proposed antenna, shedding light on its ability to minimize signal reflections and maximize power delivery. A consistently low VSWR across the operating band reflects a well-optimized design. These findings confirm the antenna’s effective impedance characteristics and reinforce its suitability for real-world communication systems. As a critical step in performance validation, the VSWR analysis confirms the design’s compliance with standard requirements and supports its practical implementation.

**Fig. 11:**
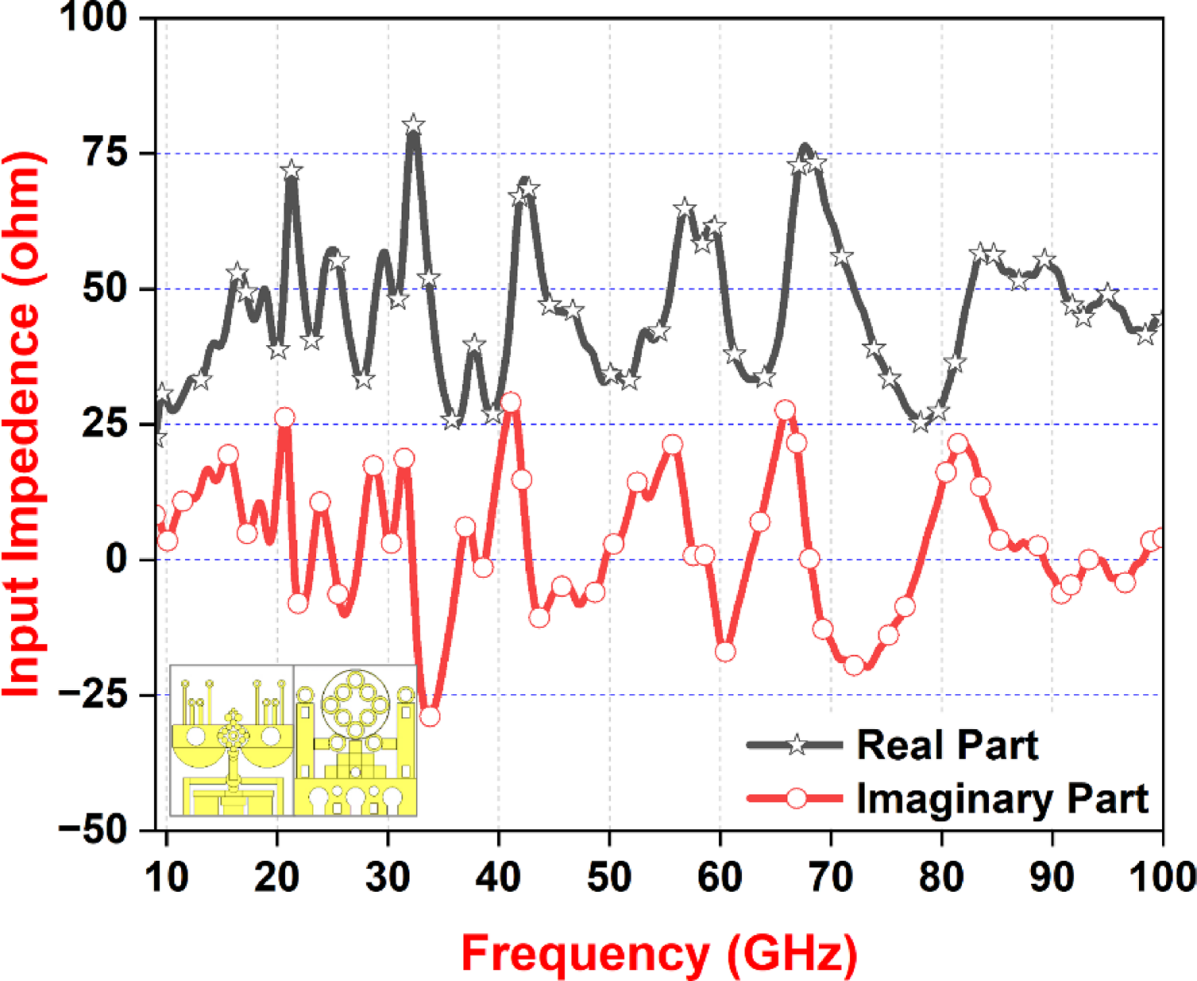
Input impedance curve.

Figure 11 shows that simulated real and imaginary components for the suggested antenna design are displayed against frequency, which depicts the input impedance curve. Optimal impedance matching is indicated by the real impedance being constantly maintained at 50 Ω throughout the intended frequency band. In the meantime, the behavior of the antenna across the frequency spectrum is shown by the imaginary impedance curve. Its positive polarity indicates that it first displays inductive characteristics, which range from 9.1 to 21 GHz. Then there is a change from 21 to 22 GHz that exhibits capacitive behavior (caused by the negative polarity). Above this threshold, as Fig. 9 shows, the behavior alternates between inductive and capacitive. This thorough analysis offers insightful information about the antenna design’s impedance properties, enabling a thorough comprehension of its performance in various frequency ranges.

**Fig. 12:**
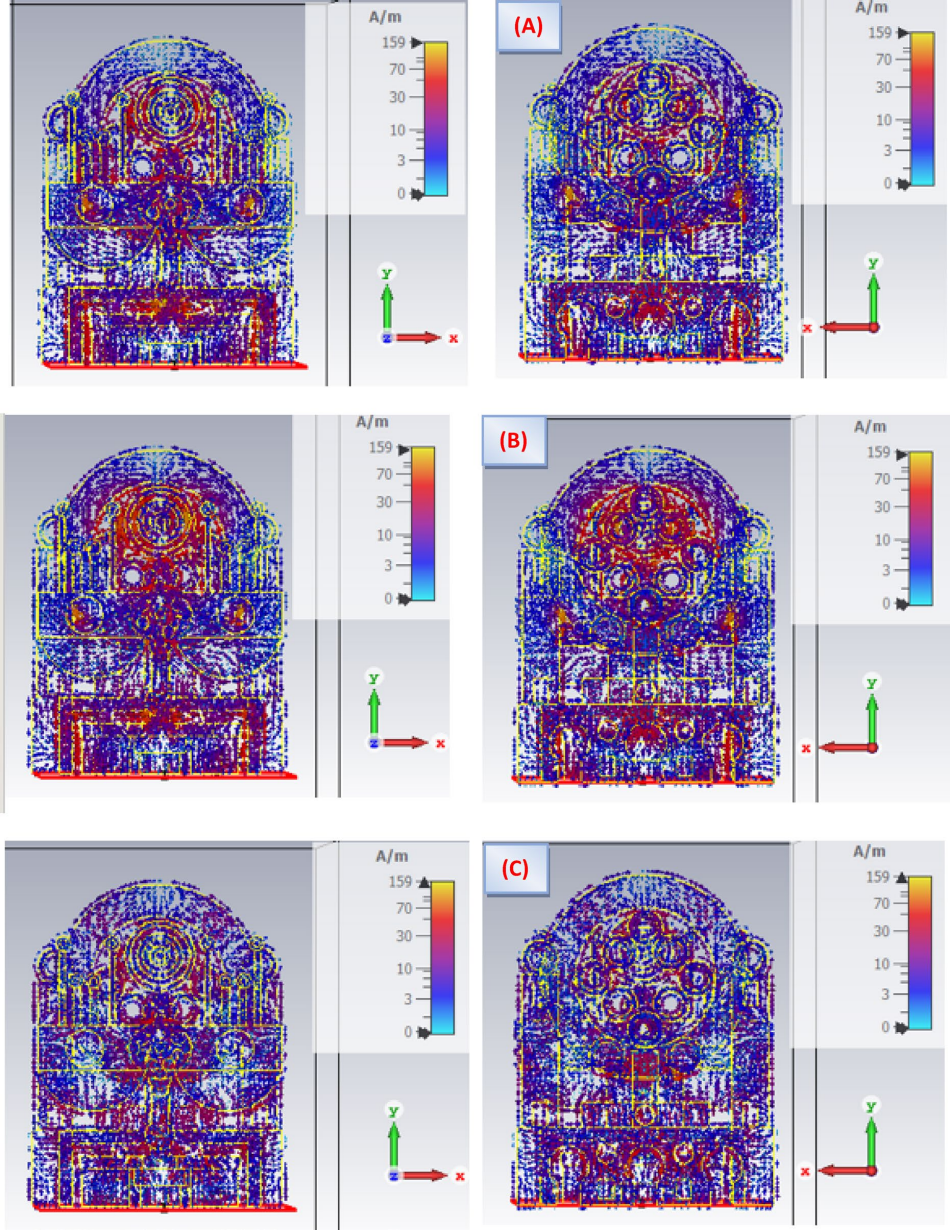
Surface current distribution of the antenna at (**A**) 19 GHz, (**B**) 20 GHz and (**C**) 40 GHz.

Figure 12 illustrates the simulated surface current distribution of the antenna at three operating frequencies: 19 GHz, 20 GHz, and 40 GHz. This visual representation highlights the intensity of current flow across the antenna’s surface, with color gradients indicating different levels of current magnitude. By analyzing these distributions, it becomes easier to identify regions with concentrated current, offering valuable insight into how different parts of the antenna contribute to radiation. The observation of both front and back surface currents at the specified frequencies provides a clearer understanding of current behavior and propagation. Such analysis is crucial for refining the antenna layout and identifying areas where performance may be compromised due to uneven current distribution. By studying how the current flows, designers can enhance efficiency, reduce losses, and optimize the antenna’s performance.

**Fig. 13:**
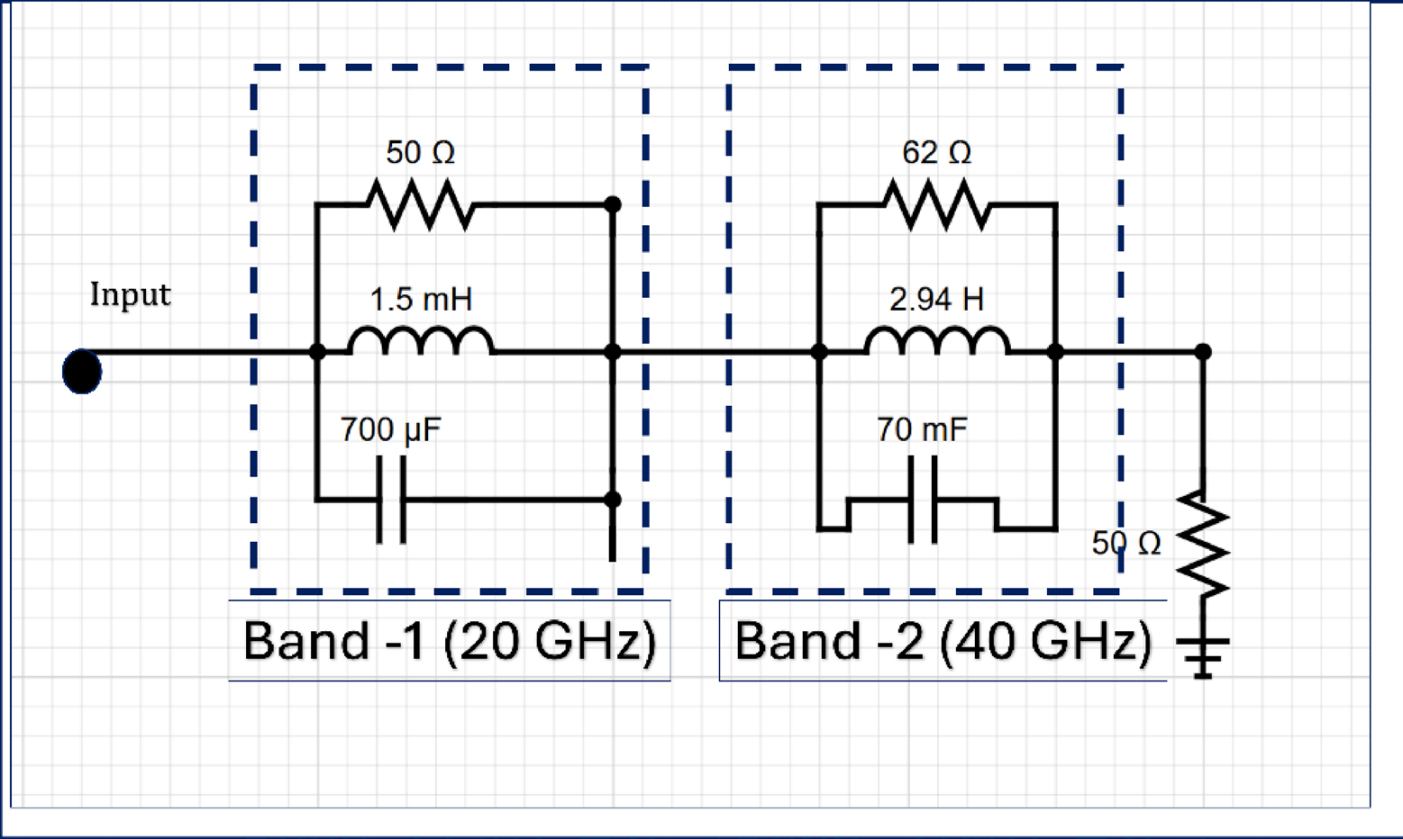
Equivalent circuit of the proposed antenna.

The equivalent circuit model, as depicted in the Fig. 13, is developed to emulate the dual-band response of the proposed system. It comprises two distinct parallel RLC resonant branches corresponding to Band-1 (centered at 20 GHz) and Band-2 (centered at 40 GHz). The first branch, representing Band-1, includes a 1.5 mH inductor, a 700 µF capacitor, and a 50 Ω resistor arranged in parallel. This configuration produces a resonance around 20 GHz, allowing efficient signal transmission at the targeted frequency while suppressing out-of-band components. Similarly, the second branch, tuned for Band-2 operation, consists of a 2.94 H inductor, a 70 mF capacitor, and a 62 Ω resistor, forming another parallel RLC circuit that resonates near 40 GHz. The values of inductance and capacitance are carefully chosen to achieve resonance at the desired frequencies. A 50 Ω resistor is connected at the output to simulate a matched load, ensuring minimal reflection and accurate characterization of the frequency response. This equivalent circuit model provides a simplified yet effective representation of the system’s frequency-selective behavior, making it a valuable tool for analyzing, optimizing, and validating the multiband characteristics of RF and millimeter-wave components.

**Fig. 14:**
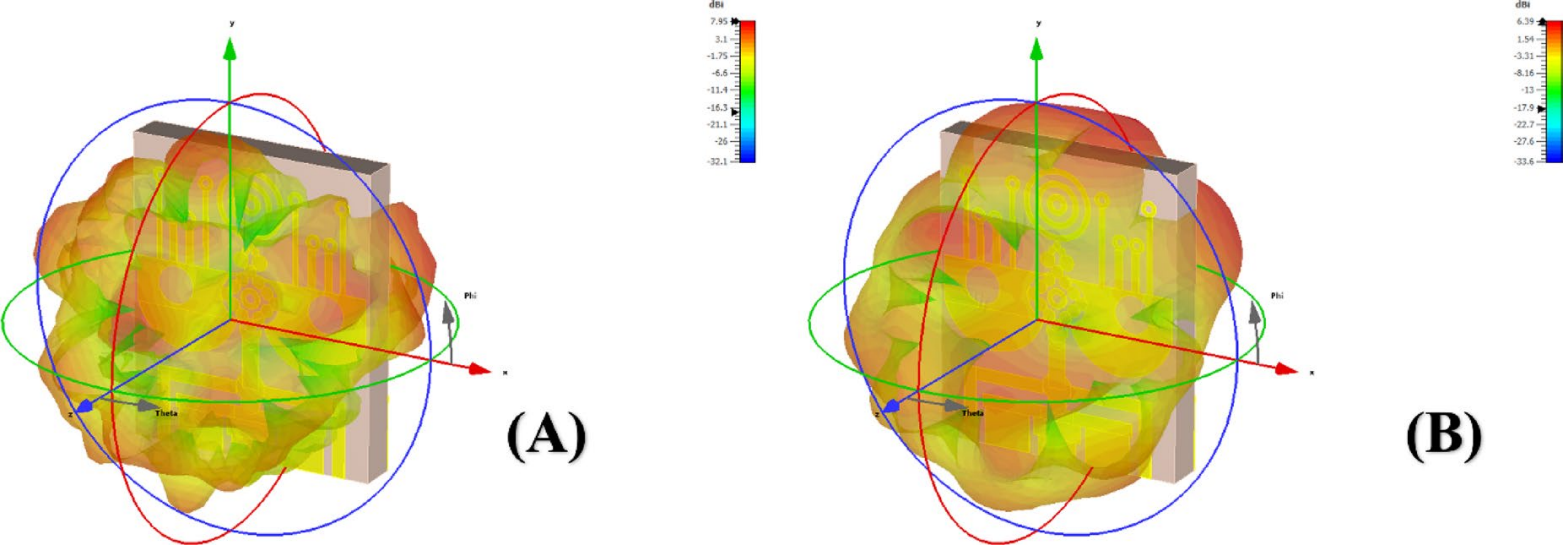
Radiation antenna lobe patterns (**a**) ‘left phase lobe’ at 20 GHz frequency and (**b**) ‘right phase lobe’ at 40 GHz frequency.

We appreciate the reviewer’s insightful suggestion. In response, we have incorporated the simulated realized gain, radiation efficiency, and 3D radiation patterns (co-polarization and cross-polarization) for the operational bands centered at 20 GHz and 40 GHz. Figure 14A,B present the simulated 3D radiation patterns of the proposed antenna at 20 GHz and 40 GHz, respectively. The plots demonstrate the spatial distribution of the radiated fields, highlighting the omnidirectional radiation characteristics across both bands. The gain color scale is provided in dBi, showing a peak simulated gain of approximately 7.95 dBi at 20 GHz and 6.39 dBi at 40 GHz. The radiation patterns exhibit stable and symmetric lobes, indicating effective radiation with minimal distortion. Additionally, the simulated radiation efficiency is observed to be above 85% across both bands, which confirms minimal ohmic and dielectric losses in the design. The co-polarization component dominates the radiated field, while the cross-polarization remains suppressed throughout the angular spectrum, indicating high polarization purity and stable performance across the intended frequency bands.

**Fig. 15:**
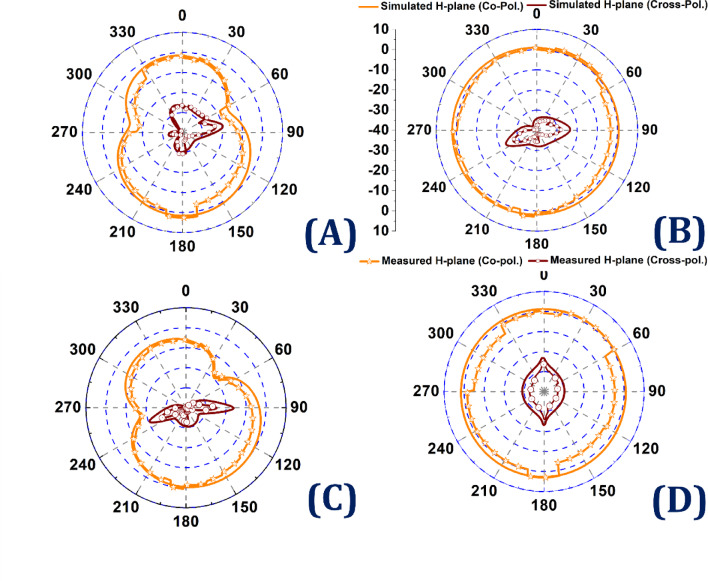
Radiation patterns of the antenna at 20 GHz and 40 GHz. (**A**,**C**) E-plane and (**B**,**D**) show the H plane.

Figure 15 presents the simulated and measured radiation patterns of the proposed antenna at 20 GHz and 40 GHz. Subfigures (A) and (C) depict the E-plane radiation patterns, whereas (B) and (D) illustrate the corresponding H-plane radiation patterns. In each plot, the co-polarized component (solid orange line) and cross-polarized component (dark red line) are shown for both simulated and measured results. The radiation patterns demonstrate excellent directional stability and uniform field distribution across both operational bands. The E-plane patterns (A and C) exhibit well-formed and symmetric main lobes with suppressed cross-polarization levels, indicating strong polarization control. In contrast, the H-plane patterns (B and D) show a quasi-omnidirectional behavior, suitable for wide-area signal coverage. The strong correlation between the simulated and measured data at both 20 GHz and 40 GHz validates the antenna’s high radiation efficiency, stable gain performance, and effective polarization purity, thus confirming its suitability for high-frequency multiband communication applications. The measured results also correlate closely with the simulated trends, validating the accuracy of the model. The slight deviations are attributed to fabrication tolerances and measurement environment variations. All the relevant plots including realized gain, radiation efficiency, and polar plots for co-pol and cross-pol components have been added in the revised manuscript under the Results and Discussion section.

The comparison Table 2 emphasizes the distinct performance differences between conventional antenna designs and proposed antennas across several key parameters.

**Table 2:** Comparison table.

Antenna	Size (mm^3^)	Impedance bandwidth (%)	Reflection coefficient (dB)	Frequency range (GHz)	Paek gain (dBi)	Efficiency (%)
Proposed antenna	60 × 70 × 1.5	70.4%	− 35 dB	4.7–67.63	9.36	89.5%
[1]	40 × 60 × 1.5	68%	− 30 dB	3.5–60	8.2	80.5%
[2]	20 × 50 × 1.6	65%	− 28 dB	4.0–50	7.8	78%
[3]	50 × 70 × 1.4	75%	− 32 dB	5.2–70	9.1	85%
[4]	30 × 55 × 1.5	62%	− 25 dB	3.5–45	7.5	78%
[5]	35 × 65 × 1.6	67%	− 31 dB	4.5–60	8.0	81%
[6]	45 × 60 × 1.5	72%	− 33 dB	4.0–65	8.8	83.5%
[7]	25 × 55 × 1.6	64%	− 26 dB	3.5–48	7.4	77%
[8]	40 × 60 × 1.5	69%	− 29 dB	5.0–55	8.5	80%
[9]	60 × 70 × 1.4	70%	− 34 dB	4.7–60	9.0	84%
[10]	20 × 50 × 1.6	68%	− 30 dB	4.0–58	8.1	81%
[11]	25 × 55 × 1.5	63%	− 27 dB	3.5–50	7.9	78.5%
Proposed	10 × 12 × 1.5	166%	− 38 dB	9.1–100	7.95	85%

## Conclusion

The paper titled “Multi-Band High-Frequency Antenna for Satellite, Automotive Radar, and 6G Communication” successfully demonstrates the design and fabrication of a compact ultra-wideband antenna optimized for millimeter-wave applications within the 5 G-II frequency range. The antenna achieves a wide impedance bandwidth of 166%, operating effectively from 9.1 to 100 GHz with a center frequency at 45.45 GHz. Featuring a peak gain of 7.95 dBi and high efficiency of 85%, the antenna delivers consistent radiation performance across multiple frequency bands. Its broad operational range makes it suitable for diverse applications such as satellite communications, automotive radar, 5G infrastructure, and emerging 6G technologies. The use of the RT/Duroid Rogers 5880 substrate and thorough electromagnetic simulation validate its potential as a reliable component for next-generation high-frequency communication systems. This work establishes a promising foundation for future advancements in ultra-wideband millimeter-wave antenna technology.

## Data Availability

The data generated during this study are included within this article.
